# Causal association between low vitamin D and polycystic ovary syndrome: a bidirectional mendelian randomization study

**DOI:** 10.1186/s13048-024-01420-5

**Published:** 2024-05-07

**Authors:** Bingrui Gao, Chenxi Zhang, Deping Wang, Bojuan Li, Zhongyan Shan, Weiping Teng, Jing Li

**Affiliations:** 1 Department of Endocrinology and Metabolism, The Institute of Endocrinology, NHC Key Laboratory of Diagnosis and Treatment of Thyroid Diseases, The First Affiliated Hospital of China Medical University, Shenyang, Liaoning 110000 P.R. China; 2 Department of Endocrinology and Metabolism, Hongqi Hospital Affiliated to Mudanjiang Medical College, Mudanjiang, Heilongjiang 157011 P.R. China

**Keywords:** Vitamin D, Polycystic ovary syndrome, Testosterone, Mendelian randomization

## Abstract

**Background:**

Recent studies have revealed the correlation between serum vitamin D (VD) level and polycystic ovary syndrome (PCOS), but the causality and specific mechanisms remain uncertain.

**Objective:**

We aimed to investigate the cause-effect relationship between serum VD and PCOS, and the role of testosterone in the related pathological mechanisms.

**Methods:**

We assessed the causality between serum VD and PCOS by using genome-wide association studies (GWAS) data in a bidirectional two-sample Mendelian randomization (TS-MR) analysis. Subsequently, a MR mediation analysis was conducted to examine the mediating action of testosterone in the causality between serum VD and PCOS. Ultimately, we integrated GWAS data with cis-expression quantitative loci (cis-eQTLs) data for gene annotation, and used the potentially related genes for functional enrichment analysis to assess the involvement of testosterone and the potential mechanisms.

**Results:**

TS-MR analysis showed that individuals with lower level of serum VD were more likely to develop PCOS (OR = 0.750, 95% CI: 0.587–0.959, *P* = 0.022). MR mediation analysis uncovered indirect causal effect of serum VD level on the risk of PCOS via testosterone (OR = 0.983, 95% CI: 0.968–0.998, *P* = 0.025). Functional enrichment analysis showed that several pathways may be involved in the VD-testosterone-PCOS axis, such as steroid hormone biosynthesis and autophagy process.

**Conclusion:**

Our findings suggest that genetically predicted lower serum VD level may cause a higher risk of developing PCOS, which may be mediated by increased testosterone production.

**Supplementary Information:**

The online version contains supplementary material available at 10.1186/s13048-024-01420-5.

## Introduction

Vitamin D (VD) is an essential fat-soluble steroid hormone that is necessary for calcium-phosphate metabolism, bone homeostasis, cell differentiation, and immune system function. The prevalence of VD deficiency (VDD) in the population has gradually increased over the past few decades. VDD is associated with various diseases, including cardiovascular disease, inflammation, dyslipidemia, weight gain, and infectious diseases [1, 2]. Furthermore, mounting studies have indicated the potential link between the serum VD status and women's reproductive health. Firstly, the biological function of VD is mediated via intracellular VD receptors (VDRs), which are distributed among various tissues, encompassing hypothalamic, pituitary tissue, endometrium, and ovary [3, 4]. Secondly, VD participates in regulating genes associated with ovarian and placental functions [5, 6]. All evidences suggest that the serum VD plays a potentially significant role in female reproductive health.

Polycystic ovary syndrome (PCOS) is the most common endocrine disorder that effects women of reproductive age, with a global incidence ranging 20–25% [7, 8]. PCOS will affect woman's endometrial function and oocyte competence [9, 10], which leads to reproductive dysfunction in PCOS patients, including infertility, miscarriage, and pregnancy complications [11–13]. However, the exact pathogenesis of PCOS remains unclear. Prior observational studies have elucidated the correlation between the serum VD and the risk of PCOS. A recent study revealed that serum VD concentration were lower in women diagnosed with PCOS compared to body mass index (BMI)-matched control, suggesting that regardless of BMI, PCOS is correlated with reduced VD level [14]. However, these studies can only prove that there is a correlation between them, they cannot clarify the causality between them. In addition, hyperandrogenemia stands as one of the diagnostic criteria for PCOS and impacts 60–80% of patients [15]. Female are actually more sensitive to testosterone even though it is known as a male hormone [16]. Growing evidences showed that testosterone may play an important role between the serum VD level and the risk of PCOS. Hahn et al. illustrated an association between the serum VD level and the severity of hirsutism in individuals with PCOS [17]. The research conducted by Latic et al. indicates a negative correlation between serum VD level and testosterone production in patients with PCOS [18]. However, a study by Mesinovic et al. suggested no discernible correlation between the serum VD level and androgen production in individuals with PCOS [19]. Moreover, a large observational study by Gallea et al. also showcased the association between serum VD levels, insulin, and body weight among PCOS patients but not specifically with hyperandrogenemia [20]. The reason for these different results may be due to the fact that observational studies are susceptible to confounding factors as well as various biases [21]. Therefore, it is not clear whether testosterone production mediate the relationship between serum VD level and the risk of PCOS, due to the limitations of the study methodology.

In recent years, mendelian randomization (MR) analysis is widely used as an epidemiological method in medical research. Firstly, MR analysis can minimize the impact of confounding factors and various biases on the results by simulating randomized controlled trials (RCTs) at the genetic level, and secondly, MR analysis can also determine causality and reduce the impact of reverse causality on the results of the study [22].

Thus, in this study, we use the bidirectional two-sample MR (TS-MR) analysis to investigate the cause-effect relationship between the serum VD level and the risk of PCOS. Secondly, we perform the mediation MR analysis to test the mediating role of testosterone production between serum VD level and the risk of PCOS. Finally, we used the bioinformatics analysis to assess the possible biological functions and molecular mechanisms between them.

## Materials and methods

### Study design of mendelian randomization study

**Fig. 1:**
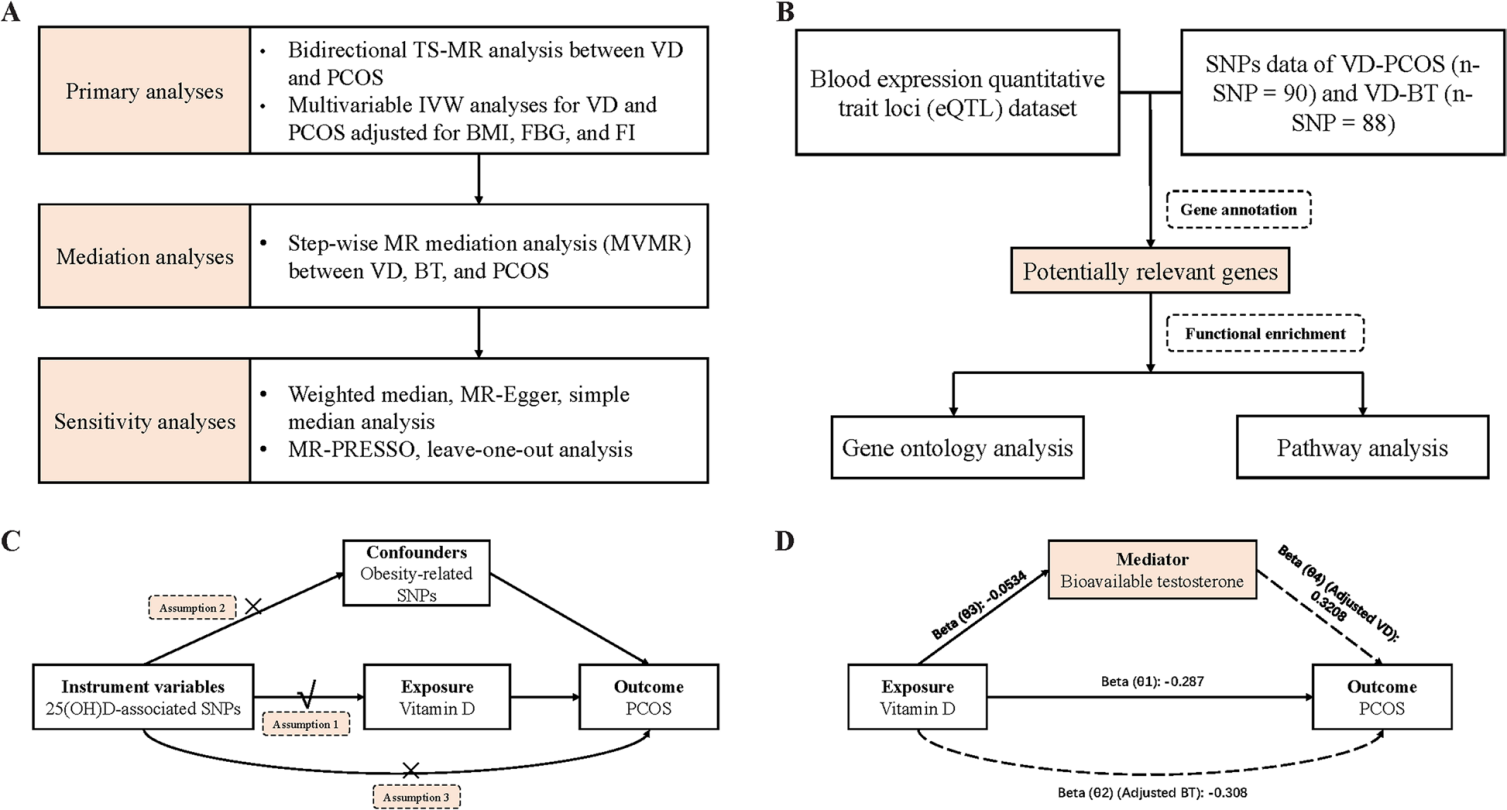
Flowchart of the study. **A** Flowchart of the MR study; (**B**) Flowchart of the Bioinformatics study; (**C**) Diagram of the MR assumptions of the association between VD and PCOS; (**D**) Illustrative diagram for the mediation MR analysis framework Abbreviations: MR, mendelian randomization; TS-MR, two-sample MR; VD, vitamin D; PCOS, polycystic ovary syndrome; IVW, inverse variance weighted; BMI, body mass index; FBG, fasting glucose; FI, fasting insulin; MVMR, multivariable MR; BT, bioavailable testosterone; SNPs, single-nucleotide polymorphisms

Our study explored the cause-effect of serum VD level as an exposure on the risk of developing PCOS as an outcome trait and the effect of testosterone as a mediator between VD and PCOS through bidirectional TS-MR analysis, multivariable MR (MVMR) and mediator MR analysis (Fig. 1). In order to ensure the study's validity, the study needed to meet the three following crucial assumptions [23] (Fig. 1C):1) the correlation assumption: instrumental variables (IVs) must be robustly correlated with the exposure factors; 2) the exclusion restriction assumption: IVs are not associated with potential confounders of the exposure or the outcome; and 3) the independence assumption: IVs do not influence the outcome variables through other pathways besides the exposure factors. This study followed guidelines of STROBE-MR [24] checklist (Table S1).

### Data source and IVs selection of mendelian randomization study

We obtained data associated with VD from a large genome-wide association study (GWAS) that identified 143 loci among 417,580 participants which was conducted by Revez et al. in 2020 [25]. We accessed the summary data related to PCOS from a meta-analysis in the FinnGen and Estonian Biobank (EstBB), which included 3609 cases and 229,788 controls [7]. Summary data related to bioavailable testosterone (BT) were obtained from the UK Biobank (UKB). Data on serum fasting glucose (FBG) levels were obtained from a UKB GWAS we conducted in 340,002 British participants [26]. Summary data on circulating concentrations of fasting insulin (FI) were obtained from the MAGIC GWAS included 151,013 participants [27]. Pooled data related to BMI were acquired from a GWAS meta-analysis within the (GIANT) consortium, encompassing 681,275 participants [28]. Details of the GWAS database are summarized in Table S2.

In the bidirectional TS-MR analysis, Single-nucleotide polymorphisms (SNPs) with genome-wide significance (*P* < 5 × 10^–8^) were first selected. These SNPs were matched against the SNP-outcome GWAS database to exclude SNPs that could not be matched. To minimize the effects of linkage disequilibrium, we conducted a clumping process with an *r*^2^ threshold of 0.001 and a clumping window of 10,000 kb and excluded these SNPs if present. Subsequently, we performed MR-PRESSO analysis immediately to demonstrate whether there was significant horizontal pleiotropy to exclude outlier SNPs [29]. To ensure that the IVs were not affected by confounding variables, we searched the PhenoScanner V2 [30] and deleted obesity-related SNPs associated with BMI and waist circumference (WC). Finally, 88 SNPs (VD on PCOS) and 2 SNPs (PCOS on VD) were used as IVs in the primary bidirectional TS-MR study, respectively. All SNPs exhibited an F statistic greater than 10. The variance explained for each SNP (R^2^) was calculated using the widely-accepted formula [31, 32]. We used the same method as above to screen the SNPs required in the MR mediation analysis. All the IVs SNPs are summarized in Table S3-7.

### Statistic analysis of mendelian randomization study

Initially, the primary analysis aimed to explore the causal relationship between VD and PCOS. We used bidirectional TS-MR analysis to assess the causal relationship between VD and PCOS. In this, we used Cochran's Q test to assess the heterogeneity [33]; if there was no heterogeneity, we would use the fixed-effects inverse variance weighted (IVW) method, otherwise, we would use the random-effects IVW method [34]. Furthermore, considering that obesity, abnormal insulin levels, and abnormal glucose values are common in patients with PCOS, we adjusted genetically predicted BMI, FBG, and FI by MVMR to explore the direct causal effect between VD and PCOS. To make the results more robust.

Secondly, a stepwise MR analysis approach was used to examine whether there exist mediation effects of BT between VD and PCOS. To assess the direct causal effect between VD, BT, and PCOS, we performed an MVMR analysis using the MVMR R package [35]. Conditional F statistics were calculated for assessing the strength of the genetic instruments in MVMR analysis [36]. The product of the coefficients method [37] and the multivariate delta method [38] were used to calculate the indirect effects of VD on PCOS via mediator.

### Sensitivity analysis of mendelian randomization study

The following tests were used as sensitivity analyses to assess the robustness of MR effect estimates to invalid genetic variants. Firstly, we conducted MR-Egger regression [39, 40], weighted median [41], and weighted mode [42] methods. MR-Egger regression can detect and explain horizontal pleiotropy mainly through intercept tests [39, 40]. Weighted median can yield impartial estimations even when over half of the information arise from flawed IVs [43]. We used weighted mode to divide SNPs into multiple subsets based on similar causal effects, and the estimates of causal effects were computed for the subset with the highest number of SNPs [42]. Secondly, the leave-one-out (LOO) analysis can test whether the results are affected by a single SNP [44]. Thirdly, as described above we performed MR-PRESSO analysis [29] to identify the presence of potential horizontal pleiotropic outliers in IVs that could lead to biased results, as well as searching for and removing obesity-related SNPs associated with BMI and WC from the PhenoScanner database [45].

All analyses were conducted using R version 4.2.0 (R Foundation for Statistical Computing, Vienna, Austria). *P* values were considered significant at 0.05.

### Bioinformatical analysis

We used the largest whole blood expression quantitative trait loci (eQTL) dataset from the eQTLGen consortium, which includes data on cis-eQTLs for 19,250 whole blood expressed genes from 31,684 individuals [46]. We combined SNPs data of VD-PCOS (*n*-SNP = 90) and VD-BT (*n*-SNP = 88) with cis-eQTLs data for gene annotation, respectively. Genes with *P* < 5*10^–8^ and FDR < 0.05 were screened as potentially relevant genes for VD-PCOS and VD-BT.

Subsequently, we used these potentially relevant genes for bioinformatics analyses, including Gene ontology (GO) and Kyoto Encyclopedia of Genes and Genomes (KEGG) analyses. GO analyses [47], including biological process (BP), molecular function (MF), and cellular composition (CC), are commonly used for large-scale functional enrichment studies. KEGG is a database that stores information about genomes, biological pathways, diseases, and drugs. We used the clusterProfiler package, org.Hs.eg.db package, and enrichplot package in the software R to perform GO and KEGG enrichment analyses of the potentially relevant genes. *P* < 0.05 for GO entries and KEGG pathways were considered significant.

## Results

### Causal effect between serum vitamin D and polycystic ovary syndrome

In our bidirectional TS-MR analysis, the number of IVs of VD on PCOS and PCOS on VD were 90 and 2, respectively. The F-statistic values for each SNP were greater than 10 (Table S3), indicating that the results were almost unaffected by weak instrumental bias. The result of fixed-effects IVW method (Cochran's Q statistic = 81.42, *P* = 0.704) indicated that genetically predicted higher level of VD led to a lower risk of developing PCOS after excluding obesity-associated SNPs (*n* = 90 SNPs, OR = 0.750, 95% CI: 0.587–0.959, *P* = 0.022) (Table 1). MR-Egger, weighted median, and weighted mode methods all obtained similar magnitude and direction to IVW method (Table 1). The scatter plot demonstrates the inhibitory effect of individual SNP on PCOS (Fig. S1). Since the MR-Egger *P*-intercept was greater than 0.05 (Table S8) and the funnel plot (Fig. S2) was roughly symmetrical, there was no indication of horizontal pleiotropy detected in the study. The results of the LOO analyses indicated that there were no potentially affecting SNPs in the main MR analyses (Fig. S3). The result of the result of the MR-PRESSO test did not show any outlier SNPs. Nevertheless, the results of reverse TS-MR showed that genetically predicted risk of developing PCOS did not affect the VD level (fixed-IVW: *n* = 2 SNPs, OR = 1.004, 95% CI: 0.987–1.022, *P* = 0.640) (Table 1).

**Table 1:** Bidirectional two-sample Mendelian randomization results of the serum VD level and the risk of PCOS

**Exposure**	**Outcome**	**SNPs, n**	**Method**	**OR (95%CI)**	**β**	***P***
VD	PCOS	90	IVW (fixed)	0.750 (0.587-0.959)	-0.287	0.022
		90	Weighted median	0.833 (0.564-1.231)	-0.182	0.359
		90	MR Egger	0.846 (0.570-1.255)	-0.168	0.407
		90	Simple median	0.748 (0.499-1.121)	-0.290	0.160
PCOS	VD	2	IVW (fixed)	1.004 (0.987-1.022)	0.004	0.640

*Abbreviations: VD* Vitamin D, *PCOS* Polycystic ovary syndrome, *SNPs* Single-nucleotide polymorphisms, *IVW* Inverse variance weighted, *NA* Not available, *OR* Odds ratio

We subsequently explored the direct effect of the serum VD level on PCOS by MVMR methods, and the results of both Model 1 (adjusted BMI) and Model 2 (adjusted BMI, FBG, and FI) showed that the negative correlation between serum VD level and the risk of PCOS remained similar (Table 2). This confirms the robustness of the TS-MR results.

**Table 2:** Multivariate mendelian randomization results of the serum VD level on the development of PCOS risk

**Method**	**OR (95%CI)**	**β**	***P***
IVW (no adjusted)	0.750 (0.587-0.959)	-0.287	0.022
Model 1 (adjusted BMI)	0.704 (0.521-0.952)	-0.351	0.023
Model 2 (adjusted BMI, FBG, and FI)	0.694 (0.510-0.945)	-0.365	0.020

*Abbreviations:* *VD* Vitamin D, *PCOS* Polycystic ovary syndrome, *BMI* Body mass index, *FBG* Fasting glucose, *FI* Fasting insulin, *OR* Odds ratio, *IVW* Inverse variance weighted

### Mendelian randomization mediation analysis

After excluding the outlier SNPs and obesity-related SNPs, MVMR analysis (adjusted BT) revealed direct causal effects of serum VD level (OR: 0.735, 95% CI: 0.552–0.978; *P* = 0.035) on the risk of developing PCOS (Table 3, Fig. 1D). In the following steps of the MR mediation analysis, we found strong evidence for a causal effect of serum VD level (β: − 0.053, *P* = 0.026) on BT (Table 3). In addition to this, we also found a causal relationship between BT and PCOS (OR: 1.378, 95% CI: 1.123–1.691; *P* = 0.002) (Table 3).

**Table 3:** Step-wise MR mediation analysis (MVMR) results between the serum VD level, BT, and the risk of PCOS

	**β**	**SE**	**OR (95%CI)**	***P***
**θ**_**1**_	-0.2871	0.1253	0.750 (0.587-0.959)	0.022
**θ**_**2**_	-0.3082	0.1461	0.735 (0.552-0.978)	0.035
**θ**_**3**_	-0.0534	0.0162	0.948 (0.905-0.994)	0.026
**θ**_**4**_	0.3208	0.1044	1.378 (1.123-1.691)	0.002
**Indirect effect (θ**_**3**_**×θ**_**4**_**)**	-0.0171	0.0076	0.983 (0.968-0.998)	0.025

Indirect effect (θ_3_×θ_4_): indirect causal effect of the serum VD level on the risk of PCOS via BT

*Abbreviations:* *VD* Vitamin D, *BT* Bioavailable testosterone, *PCOS* Polycystic ovary syndrome, *MR* Mendelian randomization, *MVMR* Multivariable MR, *OR* Odds ratio, *SE* Standard error

θ_1_: total effect of the serum VD level on the risk of PCOS

θ_2_: direct effect of the serum VD level on the risk of PCOS

θ_3_: direct effect of the serum VD level on BT

θ_4_: direct effect of BT on the risk of PCOS

Taken together, we found the potential mediation pathways between VD and PCOS: an indirect causal effect of VD on PCOS risk via BT (θ_3_ × θ_4_) (OR: 0.983, 95% CI: 0.968–0.998; *P* = 0.025) (Table 3). The pathway mediated 5.96% of the total causal effect of VD on PCOS risk. Detailed estimates of direct and indirect causal effects can be found in Table 3.

### Bioinformatics study

The results of the MR study suggested that reduced VD level may lead to the development of PCOS, and BT is a mediator between VD and PCOS, meaning that VD can ultimately influence the development of PCOS by affecting the production of testosterone. On the basis of the above studies, we collected IVs of VD-PCOS (*n*-SNPs = 90) and VD-BT (*n*-SNPs = 88) respectively, and combined these IVs with cis-eQTLs data for gene annotation respectively. Ultimately, 147 (VD-PCOS) and 164 (VD-BT) potentially relevant genes were annotated (Table S9-10), respectively. We then used these genes to perform GO and KEGG analyses.

**Fig. 2:**
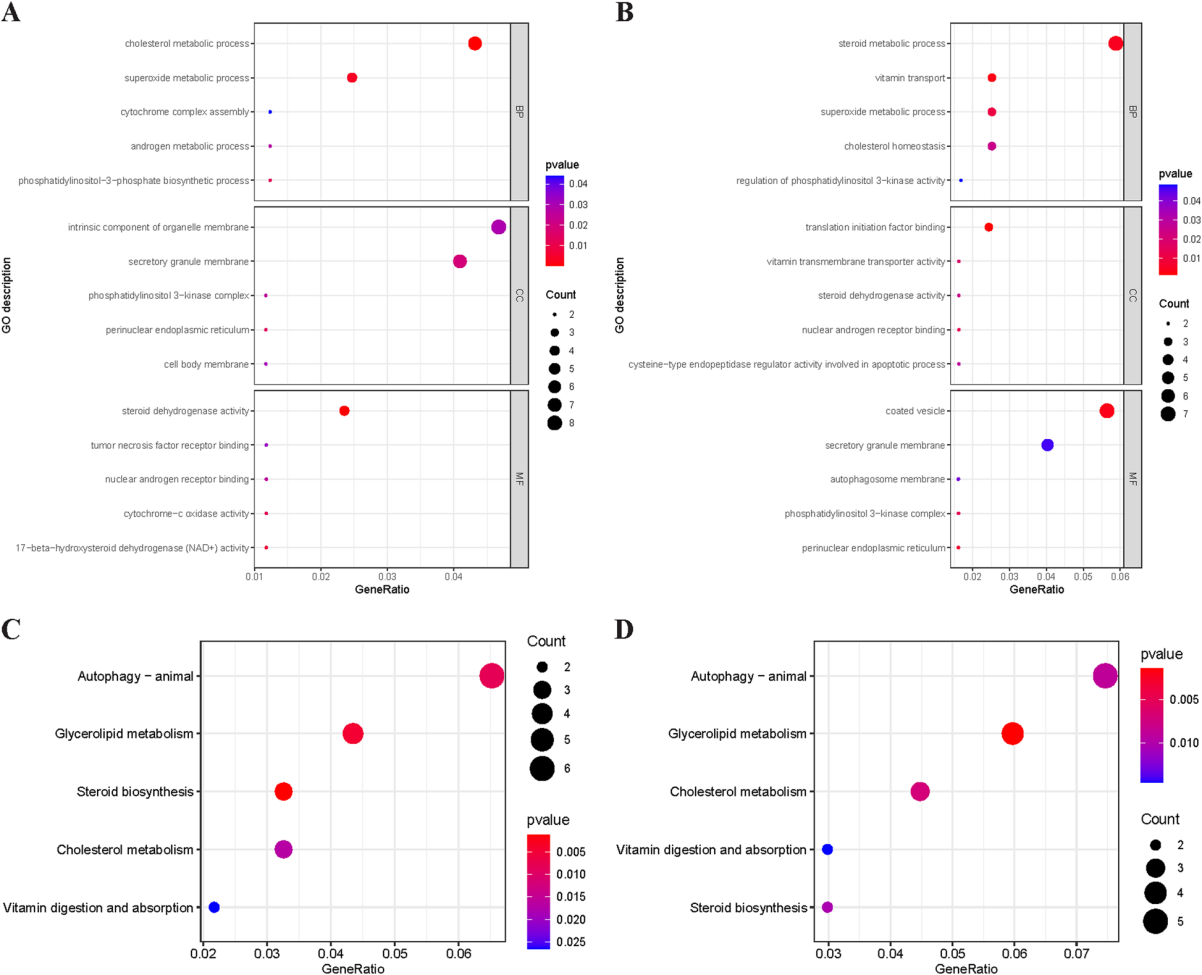
Gene Ontology and Kyoto Encyclopedia of the Genome pathway enrichment analysis of potentially relevant genes. **A** The GO enrichment analysis for potentially relevant genes related to VD and PCOS; (**B**) The GO enrichment analysis for potentially relevant genes related to VD and BT; (**C**). The KEGG pathway analysis for potentially relevant genes related to VD and PCOS; (**D**). The KEGG pathway analysis for potentially relevant genes related to VD and BT. Abbreviations: VD, vitamin D; PCOS, polycystic ovary syndrome; BT, bioavailable testosterone; GO, Gene Ontology; KEGG, Kyoto Encyclopedia of the Genome

Firstly, the potentially relevant genes of VD-PCOS were analyzed for enrichment. The results of GO analysis suggested that these genes were mainly related to androgen metabolic process, superoxide metabolic process, cell body membrane, and steroid dehydrogenase activity (Fig. 2A). The KEGG analysis was mainly enriched in the process of autophagy, steroid biosynthesis, cytochrome P450 metabolic process, and vitamin digestion and absorption process (Fig. 2C). Subsequently, potentially relevant genes associated with VD-BT were analyzed for enrichment. The results of GO analysis suggested that these genes were mainly associated with steroid metabolism, superoxide metabolism, autophagosome membrane, nuclear androgen receptor binding, and vitamin transmembrane transporter activity (Fig. 2B), and the KEGG analysis was mainly enriched for autophagy, steroid biosynthesis, vitamin digestion and absorption, and cholesterol metabolism process (Fig. 2C). All information of the enrichment analysis is shown in the additional file (Table S11-S12).

## Discussion

In our bidirectional TS-MR analysis, we found that higher serum VD level was causally associated with a lower risk of developing PCOS (OR = 0.750, 95% CI: 0.587–0.959, *P* = 0.022), whereas there was little evidence for a causal effect of the risk of PCOS on the effect of serum VD level. Furthermore, our MR mediation analysis confirmed that testosterone can act as one of the mediating factors between the causality of VD and PCOS (OR = 0.983, 95% CI: 0.968–0.998, *P* = 0.025). The mediating effect of testosterone was 5.96%. Ultimately, we utilized potentially relevant genes for GO and KEGG enrichment analysis to assess the involvement of testosterone and the potential biological and molecular mechanisms between them.

VD, a lipid-soluble vitamin, plays a pivotal role in numerous biological processes. Primarily synthesized endogenously through exposure to sunlight, it is also acquired, albeit to a lesser extent, from dietary sources [48]. VDD is considered a globally prevalent nutritional deficiency, with various studies reporting prevalence rates of 58–91% among infertile women [49]. A cross-sectional study encompassing 625 women diagnosed with PCOS and 217 control subjects revealed that Chinese women diagnosed with PCOS exhibited notably lower level of VD compared to their healthy [50]. The result from a large observational study conducted by Krul-Poel et al. similarly demonstrated significantly diminished level of VD among women within the PCOS group [51]. Recent research has demonstrated that women with PCOS exhibit lower serum concentrations of VD compared to BMI-matched controls. This implies that the level of VD is linked to PCOS irrespective of BMI [14]. Aligned with the outcomes of these observational studies, our research indicated that higher serum VD level serves as a protective factor for the risk of PCOS. To eliminate the influence of obesity as a potential confounder on the results, we excluded obesity-related SNPs in our TS-MR analysis. Subsequently, in our MVMR analyses, we adjusted for genetically predicted BMI, FBG, and FI to explore the direct causal relationship between VD and PCOS. These stringent measures significantly enhance the credibility and robustness of our findings.

The precise mechanism through which serum VD operates on PCOS remains elusive. Hyperandrogenemia stands as a pivotal diagnostic criterion for PCOS. Numerous past studies have concentrated on exploring the correlation between serum VD and hyperandrogenemia in PCOS, yet the conclusions drawn from these studies have not reached a consensus. A study conducted by Latic N et al. revealed a negative correlation between serum VD level and testosterone in PCOS patients. Additionally, Menichini et al. demonstrated a positive impact of VD supplementation (4000 IU) on total testosterone [52]. However, a study by Mesinovic et al. suggested no discernible correlation between serum VD and androgens in individuals with PCOS [19]. Moreover, a large observational study by Gallea et al. also showcased associations between serum VD level, insulin, and body weight among PCOS patients but not specifically with hyperandrogenemia [20]. The inconsistencies observed in these findings might stem from variations in race, sample sizes, seasonal disparities, and the lifestyles of the included subjects. Our study, employing Mendelian randomization, effectively mitigated the impact of sample size, seasonal fluctuations, and diverse lifestyles on the outcomes. Furthermore, our research focused solely on individuals of European ethnicity, and we excluded BMI-related SNPs when incorporating instrumental variables, thereby significantly reducing BMI's potential confounding effect on the results. These measures ensured the robustness and reliability of our findings. Our results suggest that testosterone acts as a mediator between serum VD and PCOS, implying that serum VD may potentially contribute to the development of PCOS by influencing testosterone production.

The mechanism by which serum VD ultimately contributes to the development of PCOS by affecting testosterone remains unclear, but possible explanation has been proposed. Serum VD heightens the activity of aromatase within the ovary, thereby fostering the conversion of androgens to estrogens, ultimately culminating in diminished androgens production [53]. Kinuta et al. demonstrated a marked reduction in aromatase activity within the ovaries of VDR knockout mice in contrast to the control group [54]. In addition, we performed bioinformatics analysis to explore more possible biological mechanisms. Firstly, the results of GO and KEGG analyses of potentially related genes of VD-PCOS showed that steroid biosynthetic process, androgen metabolic process, and nuclear androgen receptor binding process were the possible biological mechanisms between the causality of the serum VD level and PCOS. These results are consistent with the results of our bidirectional TS-MR analysis, demonstrating again that the serum VD can ultimately influence the development of PCOS by modulating testosterone production. Subsequently, we subjected potentially relevant genes associated with VD-BT to bioinformatics analysis. The results suggested that autophagy process and superoxide metabolism process might be the biological mechanism between serum VD and testosterone.

There are very few studies linking autophagy to PCOS, and the results of these studies suggest that the development of PCOS is closely related to the process of autophagy [55]. Texada et al. showed that autophagy can regulate steroid production by modulating cholesterol transport in endocrine cells [56]. In addition to this, the role of VD-mediated autophagy in disease has been extensively studied, and basic study by Hu et al. showed that VD can mediate the regulation of autophagy function through gastric epithelial cell VD receptors, which ultimately affects the pathogenic effects of H. pylori [57]. However, whether VD can mediate autophagy ultimately leading to PCOS remains unknown. The results of the bioinformatics study in this study suggest that autophagy is most likely one of the important mechanisms underlying the relationship between VD and PCOS.

Our study has proved that lower serum VD level causes higher prevalence of PCOS. The latter could have oocyte competence and endometrial function impaired [9, 10], but also cause a few adverse outcomes related to reproduction, such as infertility, miscarriage, and premature delivery [12, 13]. It has been found that VDD could decrease the rates of ovulation and success pregnancy in the PCOS patients, leading to less live birth [58]. In addition, It has been reported that serum VD level was independent predicting factor for live birth in the PCOS patients received ovulati0on induction [59]. Yasmine et al. have reported that endometrial thickness of PCOS patients maybe improved after VD administration [60]. A recent meta-analysis has shown that VD supplementation to PCOS women could decrease the occurrence rates of early miscarriage and premature delivery [53]. The nuclear receptor of VD (VDR) and 1,25(OH)2D3 membrane binding protein are expressed in both ovarian granulosa and theca cells [61, 62]. It has been found that VD can regulate the expression of enzymes in the VDR and ovary, ultimately regulating ovarian function [63]. One study showed that VDR mRNA was significantly less expressed in granulosa cells of the women with PCOS [64]. It may cause PCOS patients to be more sensitive to VDD. Based on the above studies and ours, serum VD level need be monitored in the female population, especially in the women of reproductive age, and timely VD administration in PCOS patients would help to improve their reproductive function and pregnancy outcomes.

Our research has several advantages. Primarily, this study confirms the direct causal relationship of the serum VD level on the risk of PCOS through the utilization of the TS-MR analysis method. This method avoids the limitation commonly found in most observational studies, thereby fortifying the reliability and validity of our finding. Secondly, we ascertain the mediating function of testosterone in the relationship between serum VD and PCOS via MR mediation analysis, thus laying the groundwork for subsequent mechanistic studies. Finally, this is the first study to combine MR studies and bioinformatics analyses together to explore causal relationship and potential functional mechanisms between serum VD level, testosterone, and the risk of PCOS, which is quite different from other studies. Nonetheless, this study also has limitations. Firstly, our study failed to capture dietary and sun exposure information that may affect serum VD level. Secondly, the use of exclusively European data in a MR analysis may not be generalizable to other ethnic populations, albeit reducing the impact of ethnicity bias on the study outcomes. Finally, the absence of relevant data prevented us from independently exploring the relationship of serum VD_2_/D_3_ with the risk of PCOS, warranting further investigation.

## Conclusions

In conclusion, our studies confirm the causality between lower serum VD level and higher risk of PCOS. Furthermore, testosterone may act as a mediator between serum VD and PCOS. These findings emphasize the clinical importance of testing serum VD level and timely VD supplementation as possible primary prevention and treatment of PCOS.

### Supplementary Information

**Additional file 1: Table S1.** STROBE-MR Checklist; **Table S2.** Key characteristics of participating studies; **Table S3.** GWAS significant SNPs used as genetic instruments for VD level on PCOS; **Table S4.** GWAS significant SNPs used as genetic instruments for PCOS on VD level; **Table S5.** GWAS significant SNPs used as genetic instruments for VD level on BT; **Table S6.** GWAS significant SNPs used as genetic instruments for BT on PCOS; **Table S7.** GWAS significant SNPs used as genetic instruments for BT and VD level on PCOS; **Table S8.** Heterogeneity and directional pleiotropy test using MR-Egger intercepts; **Table S9.** Potentially relevant genes corresponding to IVs associated with VD and PCOS; **Table S10.** Potentially relevant genes corresponding to IVs associated with VD and PCOS; **Table S11.** GO and KEGG enrichment analysis for potentially relevant genes related to VD and PCOS; **Table S12.** GO and KEGG enrichment analysis for potentially relevant genes related to VD and BT; **Figure S1.** Scatter plot of the MR estimates for the association of VD level with PCOS; **Figure S2.** Funnel plot reveals overall heterogeneity of the impact of VD on PCOS; **Figure S3.** Leave-one-out analysis of the impact of the VD on PCOS.

## Data Availability

No datasets were generated or analysed during the current study.

## Abbreviations

VD: Vitamin D
PCOS: Polycystic ovary syndrome
GWAS: Genome-wide association studies
TS-MR: Two-sample Mendelian randomization
Cis-eQTLs: Cis-expression quantitative loci
VDD: VD deficiency
VDRs: VD receptors
BMI: Body mass index
MR: Mendelian randomization
MVMR: Multivariable MR
IVs: Instrumental variables
BT: Bioavailable testosterone
UKB: UK Biobank
FBG: Fasting glucose
FI: Fasting insulin
SNPs: Single-nucleotide polymorphisms
WC: Waist circumference
IVW: Inverse variance weighted
LOO: Leave one out
GO: Gene ontology
KEGG: Kyoto Encyclopedia of Genes and Genomes
BP: Biological process
MF: Molecular function
CC: Cellular composition
