# Supplementary material for: Causal association between low vitamin D and polycystic ovary syndrome: a bidirectional mendelian randomization study
Source: J Ovarian Res. 2024 May 7;17:95. doi: 10.1186/s13048-024-01420-5 (PMC11077756; doi:10.1186/s13048-024-01420-5)
Supplement: Supplementary file 1 — Additional file 1: Table S1. STROBE-MR Checklist; Table S2. Key characteristics of participating studies; Table S3. GWAS significant SNPs used as genetic instruments for VD level on PCOS; Table S4. GWAS significant SNPs used as genetic instruments for PCOS on VD level; Table S5. GWAS significant SNPs used as genetic instruments for VD level on BT; Table S6. GWAS significant SNPs used as genetic instruments for BT on PCOS; Table S7. GWAS significant SNPs used as genetic instruments for BT and VD level on PCOS; Table S8. Heterogeneity and directional pleiotropy test using MR-Egger intercepts; Table S9. Potentially relevant genes corresponding to IVs associated with VD and PCOS; Table S10. Potentially relevant genes corresponding to IVs associated with VD and PCOS; Table S11. GO and KEGG enrichment analysis for potentially relevant genes related to VD and PCOS; Table S12. GO and KEGG enrichment analysis for potentially relevant genes related to VD and BT; Figure S1. Scatter plot of the MR estimates for the association of VD level with PCOS; Figure S2. Funnel plot reveals overall heterogeneity of the impact of VD on PCOS; Figure S3. Leave-one-out analysis of the impact of the VD on PCOS. [file 13048_2024_1420_MOESM1_ESM.zip › Figure S1-S3.docx]

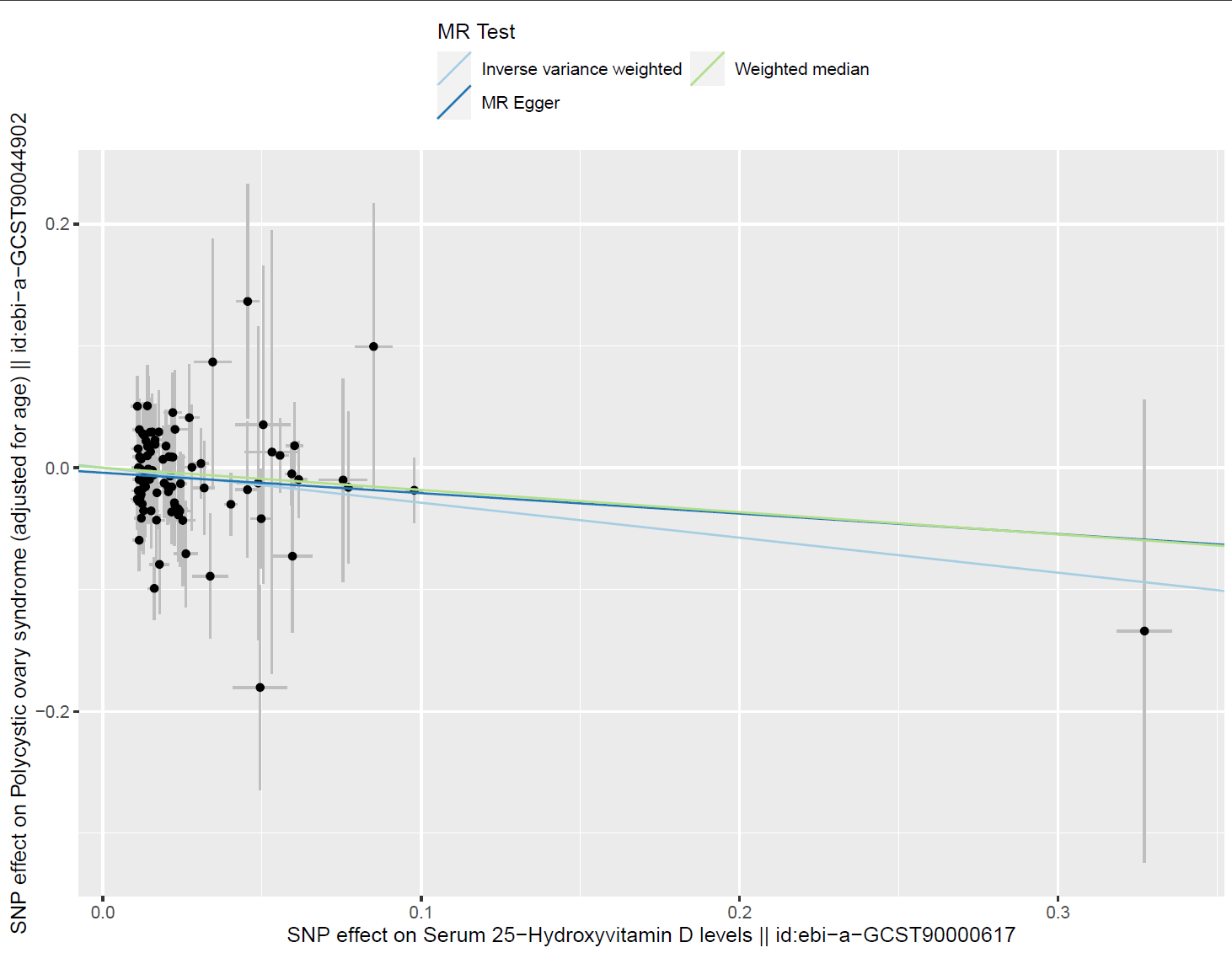


**Figure S1: Scatter plot of the MR estimates for the association of VD level with PCOS.**


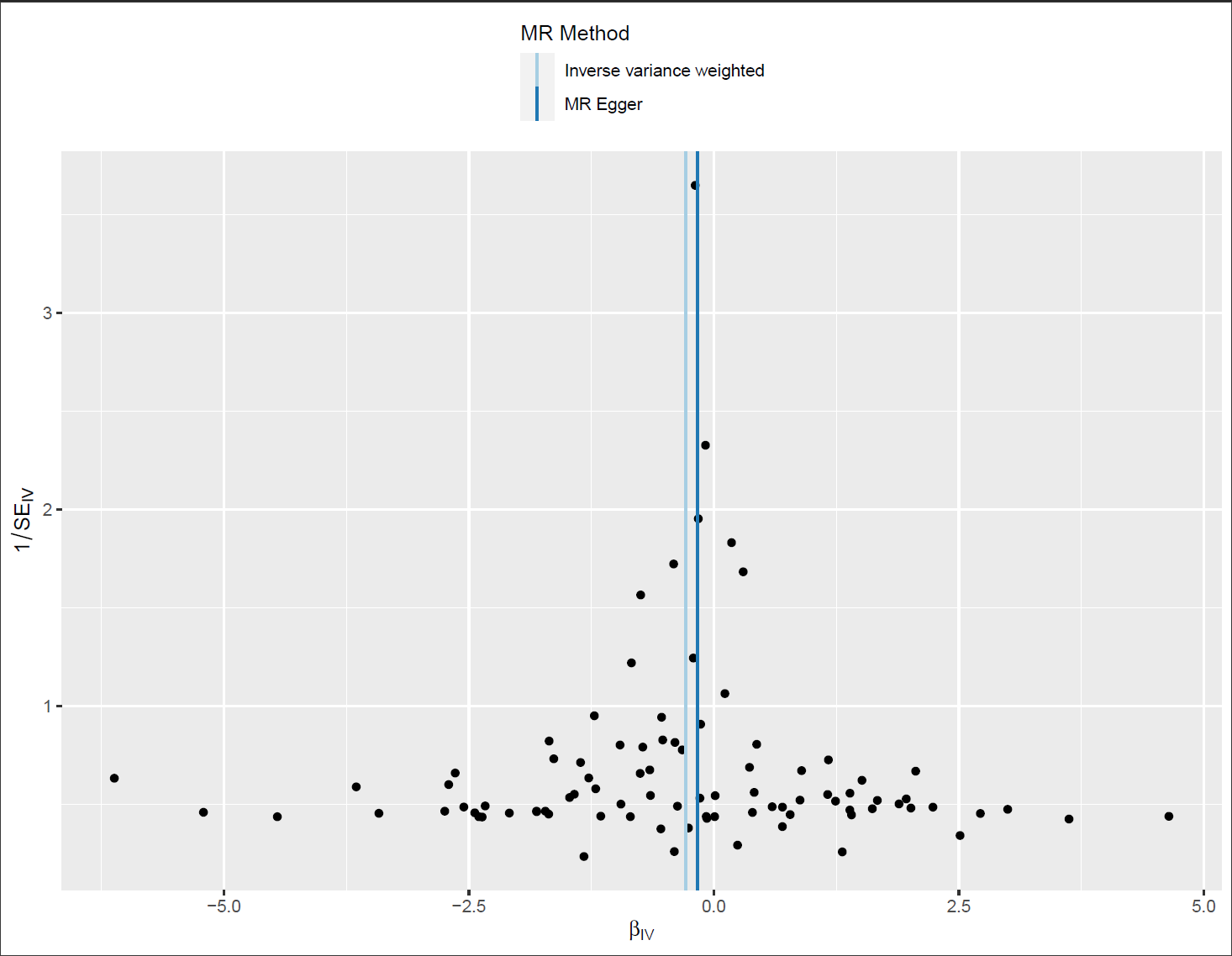


**Figure S2: Funnel plot reveals overall heterogeneity of the impact of VD on PCOS.**


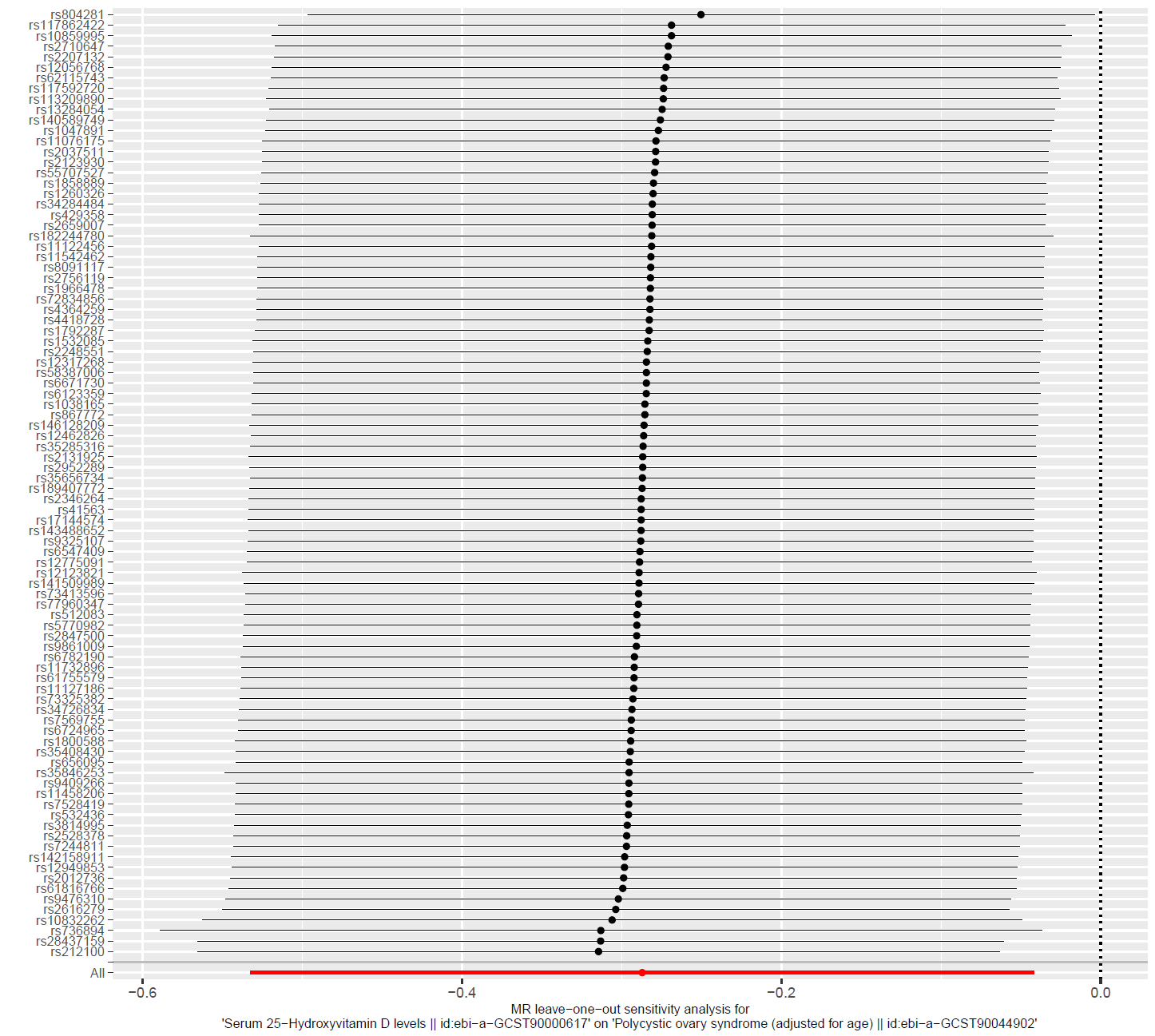


**Figure S3: Leave-one-out analysis of the impact of the VD on PCOS.**
